# Adenoide Vegetationen – Diagnostik und Therapie – die neue S2k-Leitlinie

**DOI:** 10.1007/s00106-023-01298-7

**Published:** 2023-04-18

**Authors:** Z. Ahmad, K. Krüger, J. Lautermann, B. Lippert, T. Tenenbaum, M. Tigges, M. Tisch

**Affiliations:** 1 Klinik für Hals-Nasen-Ohrenheilkunde, Kopf- und Halschirurgie, Bundeswehrkrankenhaus Ulm, Oberer Eselsberg 40, 89081 Ulm, Deutschland; 2 Institut für Allgemeinmedizin, Charité – Universitätsmedizin Berlin, Berlin, Deutschland; 3 Klinik für Hals-Nasen-Ohrenheilkunde, Kopf- und Halschirurgie, Plastische Operationen, Krankenhaus Martha-Maria Halle-Dölau, Halle-Dölau, Deutschland; 4 Klinik für Hals-Nasen-Ohrenheilkunde, Kopf- und Halschirurgie, Plastische Operationen, SLK-Kliniken Heilbronn GmbH – Standort Gesundbrunnen, Heilbronn, Deutschland; 5 Klinik für Kinder- und Jugendmedizin, Sana Klinikum Lichtenberg, Berlin, Deutschland; 6 Hals-Nasen-Ohrenklinik, Städtisches Klinikum Karlsruhe, Karlsruhe, Deutschland

**Keywords:** Hyperplasie, Adenoide, Adenotomie, Tonsilla pharyngealis, Schallleitungsschwerhörigkeit, Hyperplasia, Adenoids, Adenoidectomy, Pharyngeal tonsils, Conductive hearing loss

## Abstract

Eine Hyperplasie der Tonsilla pharyngealis ist als Erkrankung zu bewerten, wenn durch mechanische Obstruktion und/oder chronische Entzündungen des Nasenrachens Krankheitssymptome auftreten. Aus einer chronischen Tubenventilationsstörung können unterschiedliche Mittelohrerkrankungen wie Schallleitungsschwerhörigkeit, Cholesteatom und rezidivierende akute Otitis media entstehen. Während der Inspektion ist das Augenmerk auf das Vorliegen einer Facies adenoidea mit dauerhaft offenem Mund und sichtbarer Zungenspitze zu legen. Bei starken Beschwerden und/oder frustranen konservativen Therapieversuchen erfolgt die Adenotomie in der Regel ambulant. Die herkömmliche Kürettage gilt nach wie vor als etablierte Standardmethode in Deutschland. Bei klinischen Hinweisen auf Mukopolysaccharidose ist die histologische Untersuchung indiziert. Wegen des Blutungsrisikos wird auf den Gerinnungsfragebogen, der obligat vor jedem operativen Eingriff im Kindesalter durchzuführen ist, hingewiesen. Trotz ordnungsgemäßer Adenotomie kann es zu einem Rezidiv der adenoiden Vegetationen kommen. Vor Entlassung in das häusliche Umfeld sollte eine HNO-ärztliche Kontrolle durch Inspektion des Rachens auf Nachblutung und eine anästhesiologische Freigabe erfolgen.

## Abstract (en)

Hyperplasia of the pharyngeal tonsils is to be considered pathologic when nasopharyngeal symptoms of mechanical obstruction and/or chronic inflammation occur. Chronic Eustachian tube dysfunction can result in various middle ear diseases such as conductive hearing loss, cholesteatoma, and recurrent acute otitis media. During examination, attention should be paid to the presence of adenoid facies (long face syndrome), with a permanently open mouth and visible tip of the tongue. In the case of severe symptoms and/or failure of conservative treatment, adenoidectomy is usually performed on an outpatient basis. Conventional curettage remains the established standard treatment in Germany. Histologic evaluation is indicated for clinical evidence of mucopolysaccharidoses. Due to the risk of hemorrhage, the preoperative bleeding questionnaire, which is obligatory before every pediatric surgery, is referred to. Recurrence of adenoids is possible despite correct adenoidectomy. Before discharge home, otorhinolaryngologic inspection of the nasopharynx for secondary bleeding should be performed and anesthesiologic clearance obtained.

## Definition

Unter adenoiden Vegetationen ist die Hyperplasie der Rachenmandel (Tonsilla pharyngealis) zu verstehen, die in Verbindung mit einer mechanischen Obstruktion und/oder chronisch entzündlichen Prozessen im Nasenrachen steht. Entzündliche Erkrankungen der adenoiden Vegetationen werden als Adenoiditis bezeichnet. Aus adenoiden Vegetationen können unterschiedliche Veränderungen und Folgeerkrankungen, sowohl lokal (Nase, Ohr) als auch systemisch, entstehen. Da sich das Rachenmandelgewebe in der Adoleszenz natürlicherweise zurückbildet, liegt der Altersgipfel der Erkrankungen sowie die Mehrzahl der pathologischen Veränderungen im Wesentlichen im Kindesalter zwischen dem 1. und 6. Lebensjahr.

## Anatomie

Als unpaares Organ liegt die Tonsilla pharyngealis im Rachendach am Eingang zum Nasopharynx und gehört somit zum lymphatischen Rachenring (Waldeyer), der aus mukosaassoziiertem lymphatischen Gewebe (MALT) besteht und in der stark antigenexponierten Region des gesamten Pharynx und des Respirationstrakts der Immunabwehr dient [7, 39].

Durch sagittale Faltung wird die Oberfläche der Schleimhaut, die aus mehrreihigem Flimmerepithel mit eingelagerten Plattenepithelinseln besteht, stark vergrößert. Die Blutversorgung besteht aus kleineren Abgängen, die im Wesentlichen aus der A. pharyngea ascendens als Abgang der A. carotis externa gespeist werden.

## Pathologie/Ätiologie

Die Größenzunahme der Reaktionszentren des lymphatischen Gewebes und der Lymphfollikel führt pathologisch-anatomisch zu einer Hyperplasie. Als Ursache wird ein Circulus vitiosus aus Entzündung, Hyperplasie, Sekretstau und erneuter Entzündung angenommen, auch Allergien oder andere Arten der Antigenexposition können hier eine Rolle spielen [39, 47, 50].

## Pathophysiologie/Folgeerkrankungen

Wenn durch mechanische Obstruktion und/oder chronische Entzündungen des Nasenrachens Krankheitssymptome auftreten, ist die Hyperplasie der Tonsilla pharyngealis als Erkrankung zu bewerten. Durch die teilweise Verlegung beider Choanen entsteht eine Nasenatmungsbehinderung [32] sowie eine Sekretabflussstörung aus der Nase. Die Rachenmandelhyperplasie kann zu Schnarchen sowie zu einem obstruktiven Schlafapnoesyndrom führen [4, 6, 32, 36, 45]. Es werden einige Merkmale in der Literatur beschrieben, deren Auftreten auf das Vorliegen eines obstruktiven Schlafapnoesyndroms hinweisen, so besteht z. B. ein Zusammenhang zwischen Schlafstörungen und Enuresis nocturna [22].

Die Nasenatmungsbehinderung kann zu Malokklusion und Mundatmung [3, 4, 32] führen, welche in der Literatur als Kardinalsymptom („Facies adenoidea“) beschrieben wird. Durch die Nasenatmungsbehinderung können sich chronische Entzündungen des oberen Respirationstrakts sowie eine chronische Bronchitis als Folgeerkrankung ausbilden [15]. Zusätzlich kann eine Rachenmandelhyperplasie eine chronische Tubenfunktionsstörung mit ihren Folgen induzieren (s. S2k-Leitlinie 017-004: „Seromukotympanon“).

Im Verlauf der chronischen Tubenventilationsstörung entwickelt sich eine Vielzahl unterschiedlicher Mittelohrprobleme von der Retraktion über die Schallleitungsschwerhörigkeit bis hin zur Spontantyp-III-Situation oder auch ggf. zu einem Cholesteatom.

Auf Boden des Circulus vitiosus durch chronisch entzündetes Gewebe, Feuchtigkeit und Keimaszendenz über die Tuba auditiva kann sich eine rezidivierende akute Otitis media ausgestalten [32, 42]. Bei längerer Persistenz kann hieraus eine gestörte bzw. verzögerte Sprachentwicklung resultieren [22, 32, 40].

Die allgemeine Entwicklung der Kinder ist häufig zusätzlich durch Gedeihstörungen, nächtliches Schnarchen und insbesondere durch Schlafstörungen mit und ohne Obstruktion gefährdet [14, 17, 18, 40].

## Symptome

Zu den typischen Symptomen einer Rachenmandelhyperplasie werden gezählt: Nasenatmungsbehinderung, chronische Mundatmung, schleimig-eitrige Rhinorrhö, gehäufte Infektanfälligkeit, wiederkehrende Infekte der oberen Atemwege, Schnarchen, Schallleitungsschwerhörigkeit, rezidivierende Mittelohrentzündungen (bis hin zum Cholesteatom) und eventuell auch Zahnfehlstellungen. Zudem sollte im Rahmen der Anamnese eine Evaluation zu nächtlichen Atemaussetzern, Schlafstörungen, Tagesmüdigkeit, auffälliger Sprachentwicklung sowie einer chronischen Bronchitis erfolgen.

## Differenzialdiagnose

Erkrankungen wie eine ausgeprägte Hyperplasie der Tonsillae palatinae und eine inkomplette Choanalatresie können ein ähnliches Beschwerdebild zeigen. Zudem können endonasale Fremdkörper, Nasenmuschelhyperplasien und infektiöse sowie allergische Rhinitiden zu Nasenatmungsbehinderungen führen.

Gutartige und vor allem bösartige Neubildungen müssen ausgeschlossen werden. Hierbei sollte insbesondere bei männlichen Jugendlichen an ein juveniles Nasenrachenfibrom (gutartiger, gefäßreicher, mit Knochendestruktion verdrängend wachsender, leicht blutender, glatter und derber Tumor) gedacht werden. Im Erwachsenenalter hingegen sind vor allem Karzinome und Lymphome hiervon abzugrenzen, die üblicherweise mit Ulzerationen, Blutungen, schmierigen Belägen, Größenzunahme sowie Schallleitungsstörungen symptomatisch werden können.

An eine Tornwaldt-Zyste, einen kugeligen geformten und mit glatter Schleimhaut überzogenen Tumor des Nasenrachens, ist ebenfalls differenzialdiagnostisch zu denken.

## Diagnostik

### Anamnese

Im Rahmen der allgemeinen und spezifischen Anamnese sollte nach Nasenatmungsbehinderung, nächtlichen Atemaussetzern, Schlafstörungen, rezidivierenden Atemwegsinfekten, bronchopulmonalen Beschwerden, Hörstörungen, Auffälligkeiten in der sprachlichen Entwicklung und allergischen Symptomen gefragt werden. Unter rezidivierenden Infekten sind hier ungewöhnlich schwer verlaufende oder zahlenmäßig deutlich über dem Altersdurchschnitt liegende Infektionen zu verstehen. Im kinder- und hausärztlichen Versorgungsbereich soll bei o. g. Anamnese und/oder auffälligen Befunde in der Otoskopie eine HNO-fachärztliche Überweisung erfolgen.

### Fachärztliche Untersuchung

Während der Inspektion ist primär das Augenmerk auf das Vorliegen einer Facies adenoidea zu legen, typischerweise mit dauerhaft offenem Mund sowie sichtbarer Zungenspitze. Zusätzlich weist häufig ein Naseneingangsekzem darauf hin [2]. Fachspezifisch ist die Rhinoskopie, sofern möglich und tolerabel, die Inspektion des Nasopharynx, idealerweise flexibel endoskopisch, die Evaluation der Gaumenmandeln, die Untersuchung auf Lymphknotenschwellungen und die Ohrmikroskopie beidseits durchzuführen. Eine Malokklusion, Zahnfehlstellungen und ein hoher Gaumen können auf eine Rachenmandelhyperplasie hindeuten.

Eine Palpation des harten/weichen Gaumens sollte präoperativ durchgeführt werden, um eine submuköse Spalte ggf. identifizieren zu können.

### Funktionsdiagnostik

Um die Mittelohrbelüftung zu beurteilen, wird eine Tympanometrie durchgeführt. Ergänzend kann bei Bedarf zusätzliche Hördiagnostik, wie insbesondere eine Schwellenaudiometrie, transitorisch evozierte otoakustische Emissionen (TEOAE) und, soweit möglich und infrastrukturell vorhanden, eine Tubenmanometrie durchgeführt werden.

### Ergänzende Diagnostik

Um präoperativ die pathophysiologischen Veränderungen, insbesondere gegenüber einer Rhinosinusitis oder Adenoiditis zu unterscheiden, erfolgt die transnasale Endoskopie mit starrer oder idealerweise flexibler Optik, soweit von den Kindern toleriert. Transoral kann die Untersuchung mit der 70°-Optik erfolgen. Ist die Untersuchung aufgrund fehlender Kooperation nicht möglich, ist die klinische Beurteilung wie insbesondere die Anamnese und ein typischer Trommelfellbefund ausreichend. Bei einer malignitätssuspekten Neoplasie oder juvenilem Nasenrachenfibrom ist zusätzlich eine differenzierte, ggf. auch umfassende bildgebende Diagnostik einzuleiten.

Weist die Anamnese darauf hin, so ist eine allergologische Diagnostik unter Einschluss inhalativer Allergene indiziert [10, 33, 49].

Entsteht aus der Anamnese der Verdacht einer Sprachentwicklungsstörung, ist eine weitere Abklärung insbesondere des Gehörs und der Sprachentwicklung geboten.

Darüber hinaus ist bei entsprechender Anamnese auch weiterführende Schlafapnoediagnostik im Einzelfall sinnvoll.

### Gerinnungsdiagnostik

In einer interdisziplinären Stellungnahme haben diverse Gesellschaften klinischer Fachbereiche die präoperative Beurteilung von Gerinnungsstörungen mittels strukturiertem Fragebogen empfohlen [53], da in verschiedenen Untersuchungen Gerinnungsstörungen anhand von Routinelaborparametern (PTT, Quick) vor dem Eingriff nicht suffizient erkannt werden konnten. So soll präoperativ bei anamnestischer und/oder familiär gehäufter Blutungsneigung eine entsprechende Gerinnungsdiagnostik durchgeführt werden. Bei gerinnungsanamnestischer Unauffälligkeit im strukturierten Fragebogen kann auf eine laborchemische Analyse der Blutgerinnung vor einer Adenotomie oder Tonsillektomie/Tonsillotomie im Kindesalter verzichtet werden. In Fällen von Sprachbarrieren ist der Einsatz des Fragebogens möglicherweise begrenzt. Hier kann die laborchemische Analyse der Blutgerinnung indiziert sein.

## Konservative Therapie

Bei alleiniger Adenoidhyperplasie ohne weitere Symptome kann eine konservative Behandlung im Sinne eines beobachtenden Abwartens durchgeführt werden. Zusätzlich gibt es Hinweise, dass eine Gabe von intranasalen Kortikoiden „off-label“ [8] einen positiven Effekt auf die Adenoidhyperplasie haben kann.

Zudem ist ein konservativer Behandlungsansatz bei relativer Kontraindikation (submuköse Gaumenspalte, Blutungsneigung) kritisch zu prüfen.

Aufgrund fehlender aktueller klinischer Studien kann keine Empfehlung für eine medikamentöse Therapie abgegeben werden. Daher sollten systemische Steroide, Antibiotika oder Antihistaminika zur Behandlung von adenoiden Vegetationen nicht eingesetzt werden.

## Operative Therapie

Bei starken Beschwerden (rezidivierende Infektneigung mit Fieber, persistierende Ohrproblematik) und/oder frustranen konservativen Therapieversuchen (zuwarten, topische Kortisone, antiallergische Behandlung) erfolgt die Adenotomie (AT), bei Seromukotympanon häufig in Verbindung mit einer Parazentese und/oder der Einlage von Paukenröhrchen (s. S2k-Leitlinie 017-004: „Seromukotympanon“). Darüber hinaus kann sie selbstverständlich auch zusammen mit Eingriffen anderer Fachgebiete (Zirkumzision, Zahnextraktion usw.) durchgeführt werden. Bei präoperativ diagnostizierter submuköser Gaumenspalte soll die Op.-Indikation besonders streng interdisziplinär mit den Kolleg*innen der Mund-Kiefer-Gesichts-Chirurgie wie auch Phoniatrie und Pädaudiologie überprüft werden.

### Indikation

Anlehnend an die derzeit aktuellen Empfehlungen der American Academy of Otolaryngology & Head and Neck Surgery (AAOHNS) und hierzu ergänzend Erkenntnissen aus der aktuellen Literatur können eine oder mehrere der folgenden Indikationen eine Adenotomie bei Rachenmandelhyperplasie begründen:

1. Innerhalb der vergangenen 12 Monate bei Kindern unter 12 Jahren 4 oder mehr Episoden einer wiederkehrenden eitrigen Rhinorrhö [44]
2. Bestehende Symptome einer Adenoiditis nach zwei antibiotischen Behandlungen. Davon sollte eine Therapie mit einem Beta-Laktamase-stabilen Antibiotikum für mindestens 2 Wochen erfolgt sein. Bei einer Rezidiventzündung sollte eine Erregerdiagnostik angestrebt werden
3. Schlafstörungen mit Nasenatmungsbehinderung, die mindestens seit 3 Monaten vorliegen: obstruktives Schlafapnoesyndrom (OSAS), sekundäre Enuresis nocturna [9, 13, 22, 27, 52]
4. Geschlossenes Näseln, Hyponasalität
5. Otitis media mit Paukenerguss seit über 3 Monaten oder bei gleichzeitig vorliegender Paukendrainage
6. Malokklusion oder orofaziale Wachstumsstörung, die kieferorthopädisch und/oder zahnärztlich dokumentiert ist
7. Kardiopulmonale Komplikationen, inklusive Cor pulmonale, pulmonale Hypertonie und Rechtsherzhypertrophie einhergehend mit oberer Atemwegsobstruktion [54]
8. Rezidivierende akute und chronische Otitis media mit Paukenerguss bei einem Alter ab 4 Jahren oder älter [30, 39, 40]
9. Chronisch rezidivierende Belüftungsstörungen des Mastoids im Sinne einer akuten und/oder chronischen Mastoiditis oder rezidivierende Mittelohrentzündungen oder Tubenbelüftungsstörungen mit Retraktion des Trommelfells
10. Sekundäre Symptome wie die Facies adenoidea [26, 39, 62]

Quelle: https://www.entnet.org/resource/clinical-indicators-adenoidectomy/ (abgerufen am 21.04.2021)

Die Dringlichkeit des Zeitpunkts der Operation ist in Abhängigkeit vom Beschwerdebild mit den beteiligten Fachdisziplinen festzulegen.

In der Literatur konnte der Vorteil der chirurgischen Behandlung bei entzündlichen sekundären Symptomen von adenoiden Vegetationen (nasale Obstruktion, Tubenventilationsstörung, rezidivierende akute Otitis media, Paukenerguss) nachgewiesen werden [26, 31, 35, 51].

Bei rezidivierenden Entzündungen der oberen Atemwege mit weniger stark ausgeprägten Symptomen konnte hingegen in randomisierten multizentrischen Studien für eine Adenotomie keine signifikante Effektivität im Vergleich zu einem beobachtenden Abwarten nachgewiesen werden [50]. Auch die früher häufig geäußerte Hypothese, Adenoide seien als mechanisches Abflusshindernis der Ohrsekretion über die Tube auditiva zu sehen, ist weder studientechnisch bis heute belegt, noch lässt sich hieraus indirekt aus der aktuellen Literatur ein harter Hinweis ableiten. Die Indikation zur Durchführung einer Adenotomie ist vor diesem Hintergrund daher heutzutage kritisch zu überprüfen.

### Ambulant/stationär

Der operative Eingriff der Adenotomie sowohl mit als auch ohne Parazentese bzw. Paukendrainage wird in der Regel ambulant vorgenommen. In Ausnahmefällen können soziale Faktoren, wie lange Anfahrtswege oder eine fehlende Betreuungsmöglichkeit zu einer stationären Behandlung führen [54]. Bei Vorliegen dokumentierter Risikofaktoren (z. B. Anfallsleiden, Mehrfachbehinderung, Asthma bronchiale, Gerinnungsstörungen) ist eine stationäre Behandlung zu empfehlen. Hier kann es im Einzelfall hilfreich sein, im Vorfeld eine entsprechende Kostenübernahmeerklärung einzuholen. Grundsätzlich regeln in Deutschland die G‑AEP-Richtlinien (German Appropriate Evaluation Protocol) die Kriterien für die Beurteilung einer erforderlichen stationären Behandlung.

### Altersbegrenzung

Die Adenotomie ist ein häufiger Eingriff im Kleinkindes- sowie Kindesalter [46]. Ab dem 6. Lebensjahr erfolgt eine physiologische Involution der Rachenmandel [62], die bis zur Pubertät abgeschlossen ist, sodass später eine Indikation zur operativen Behandlung seltener zu stellen ist. Zwingend notwendig ist jedoch eine ausgedehnte Abklärung bei einer Raumforderung im Nasopharynx, insbesondere hinsichtlich einer malignen Raumforderung [44]. Insbesondere bei rezidivierenden Tubenbelüftungsstörungen im Jugend- und Erwachsenenalter ist hier eine umfangreiche Abklärung, ggf. auch unter Einschluss einer Probenbiopsie und Bildgebung, indiziert.

### Op.-Technik

Die klassische Adenotomie wird in Deutschland mittels instrumenteller Kürettage des Nasopharynx durchgeführt. Der Eingriff wird in Rückenlage nach orotrachealer Intubation bzw. unter Einsatz supraglottischer Beatmungsmöglichkeiten in Reklination des Kopfes durchgeführt. Ein Mundsperrer wird eingesetzt, und es erfolgt die Velotraktion des Gaumensegels [28].

Als Alternativen zu der konventionellen Kürettage sind weitere Operationsverfahren wie Elektrochirurgie, Microdebriding und Radiofrequenzchirurgie verbreitet und weisen in Studien teilweise auf eine Überlegenheit der angewandten Methode, insbesondere hinsichtlich des intraoperativen Blutverlusts, des Anteils von Residuen oder postoperativen Komplikationsraten hin [1, 25, 34, 37, 60]. Andere Studien [5, 16] konnten jedoch keinen klinisch signifikanten Unterschied zwischen endoskopisch-gestützten Verfahren und der konventionellen Kürettage feststellen, sodass die herkömmliche Kürettage nach wie vor als etablierte Standardmethode in Deutschland zu bezeichnen ist.

### Histologische Untersuchung

Es gibt in Deutschland keinen nationalen Konsensus für die Indikation zur histologischen Untersuchung nach einer Adenotomie [58]. So zeigte zuletzt eine nichtrepräsentative Umfrage (unter 68 HNO-Abteilungen in Deutschland), dass 54 % der Befragten bei Kindern routinemäßig eine histologische Untersuchung zwar veranlassen, aber weniger als ein Drittel die Notwendigkeit hierfür als gegeben sieht [21].

Bei anamnestischen Hinweisen auf Tumoren sowie prä- und intraoperativen makroskopischen Auffälligkeiten ist eine histologische Untersuchung zwingend erforderlich [43]. Eine zwingende Empfehlung zur histologischen Untersuchung bei unauffälligem prä- und intraoperativem Befund kann auf Basis der derzeitigen Datenlage nicht ausgesprochen werden.

Eine Mukopolysaccharidose kann bei der histologischen Untersuchung sicher erkannt werden, bei entsprechenden klinischen Hinweisen ist daher eine Untersuchung indiziert [23].

### Postoperative Betreuung

Bei ambulanter operativer Entfernung der Rachenmandel ist nicht nur ein HNO-ärztliches postoperatives Monitoring, sondern auch eines nach anästhesiologischer Maßgabe erforderlich [55]. Bei gutem Allgemeinzustand sowie ohne Komplikationen (Schwellung, Nasenblutung, Atemnot, Narkoseüberhang) kann die Entlassung in die Betreuung von Sorgeberechtigten bzw. von Begleitpersonen erfolgen. Für 24 h muss dabei eine fortwährende Betreuung sowie Überwachung gewährleistet sein [56]. Empfehlenswert und lediglich auf langjähriger klinischer Erfahrung basierend ist eine körperliche Schonung (bei Kindergartenbesuch, Kita-Besuch, Schulbesuch für 3 Tage sowie bei Schul- und Freizeitsport für 7 Tage). In den wenigen zu dieser Fragestellung vorliegenden Studien wird ein Zusammenhang zwischen körperlicher Belastung und Nachblutungs- oder Komplikationsrate nach operativer Entfernung der Adenoide vermutet, allerdings konnte kein harter Nachweis erbracht werden [29, 61].

In allen Fällen einer Nachblutung ist eine ärztliche Vorstellung zur Abklärung und Prüfung einer ggf. notwendigen Intervention dringend angeraten.

Eine Antibiotikaprophylaxe ist grundsätzlich nicht notwendig.

### Operationskomplikationen und -folgen

Als wichtigste Komplikation der Adenotomie gilt die Blutung bzw. die Nachblutung nach erfolgter Adenotomie. Analog zur Tonsillotomie und Tonsillektomie werden die Nachblutungen in primäre (innerhalb der ersten 24 h auftretende Nachblutungen) und die sekundären (nach 24 h postoperativ hinaus auftretende Nachblutungen) eingeteilt. Nachblutungsraten divergieren in Literaturangaben zwischen 0,5 und 8,0 % [11, 38, 44, 46, 48, 59]. Auch wenn die Adenotomie als häufigster HNO-ärztlich-chirurgischer Eingriff im Kindesalter ein sicherer und seit Jahrzehnten etablierter Eingriff ist, kann es in Ausnahmefällen zu schwerstwiegender Blutungskomplikation kommen. Es sind in der Literatur einzelne Fallberichte über tödliche Nachblutungen bei zugrunde liegenden Gerinnungsstörungen sowie bei Verletzungen großer arterieller Gefäße, z. B. bei fehlender knöcherner Abdeckung oder atypischem Verlauf, publiziert [12, 19, 57]. Insbesondere hinsichtlich des Blutungsrisikos wird daher nochmals auf den Gerinnungsfragebogen, der obligat vor jedem operativen Eingriff im Kindesalter durchzuführen ist, hingewiesen [53].

Zusätzlich sind Zahnschädigungen durch Einsetzen des Mundsperrers und Verletzung des Naseneingangs, inklusive Verletzung des Knorpels, durch den Silikonschlauch im Rahmen der Velotraktion möglich, zudem kann eine postoperative Wundinfektion eine antibiotische Therapie erfordern [28, 46]. Verletzung der Uvula und Schädigungen der pharyngealen Tubenostien mit anschließenden Tubenbelüftungsstörungen und schwerwiegenden Mittelohrproblemen sind seltene Komplikationen, und diese Risiken können durch Operationen unter spiegeloptischer Sicht häufig verhindert werden.

Trotz ordnungsgemäßer Adenotomie kann es zu einem Rezidiv der adenoiden Vegetationen kommen. Dies kann im Verlauf einen weiteren Eingriff nach sich ziehen.

In der Literatur sind darüber hinaus extrem seltene Komplikationen der dauerhaften Rhinophonia (aperta und clausa), permanenter Gaumensegelinsuffizienz sowie Choanalverschluss mit Narbenbildung beschrieben [44].

Bei intensiven Koagulationen im Nasenrachenraum kann zur Vermeidung eines Grisel-Syndroms eine antibiotische Behandlung über 5 Tage erwogen werden. Es sind Fallberichte über das Grisel-Syndrom (Synonym: Watson-Jones-Krankheit oder Torticollis atlantoepistrophealis) [20] oder die deszendierende Mediastinitis [41] beschrieben. In sehr seltenen Ausnahmefällen kann es durch Einsetzen des Mundsperrers zu einer in der Regel passageren Hypoglossus-Schädigung oder auch zu Geschmacksbeeinträchtigungen durch Irritation von Glossopharyngeus-Fasern kommen. Dies sollte im Aufklärungsbogen berücksichtigt werden.

Eine temporäre Rhinophonia clausa und eine für 2–3 Tage anhaltende Odynophagie können Folgen einer Adenotomie darstellen. Zudem kann eine Schleimhautschwellung zu einer vorübergehenden Nasenatmungsbehinderung sowie zu Tubenbelüftungsstörungen mit Paukenerguss führen. Eine temporäre funktionelle Beeinträchtigung des Gaumensegelverschlusses kann zu einer nasalen Regurgitation von festen Speisen und Flüssigkeiten führen [24].

### Verlaufskontrolle

Vor Entlassung in das häusliche Umfeld sollte eine HNO-ärztliche Kontrolle durch Inspektion des Rachens auf Nachblutung und eine anästhesiologische Freigabe erfolgen. Im weiteren Verlauf sollte in den anschließenden Tagen eine fachärztliche Kontrolle des Lokalbefundes erfolgen (befundabhängig) und über eine zusätzliche begleitende Behandlung (z. B. Fortführung der Schmerztherapie) entschieden werden.

Nach Abheilung des Lokalbefundes ist bei präoperativ bestehendem Hörverlust und/oder Mittelohrbelüftungsstörung eine ambulante Untersuchung mit Kontrolle der Trommelfelle, Tympanometrie, Hörtest bzw. otoakustischen Emissionen angeraten.

## References

[1] Ark N, Kurtaran H, Ugur KS (2010). Comparison of adenoidectomy methods: examining with digital palpation vs. visualizing the placement of the curette. Int J Pediatr Otorhinolaryngol.

[2] Arnold W, Ganzer U (2011). Adenoide Vegetationen.

[3] Belcher R, Virgin F (2019). The role of the adenoids in pediatric chronic rhinosinusitis. Med Sci.

[4] Bettadahalli V, Chakravarti A (2017). Post-adenoidectomy quality of life in children with refractory chronic rhinosinusitis. J Laryngol Otol.

[5] Bhandari N, Don DM, Koempel JA (2018). The incidence of revision adenoidectomy: a comparison of four surgical techniques over a 10-year period. Ear Nose Throat J.

[6] Bonuck K, Rao T, Xu L (2012). Pediatric sleep disorders and special educational need at 8 years: a population-based cohort study. Pediatrics.

[7] Brandtzaeg P (2011). Potential of nasopharynx-associated lymphoid tissue for vaccine responses in the airways. Am J Respir Crit Care Med.

[8] Chohan A, Lal A, Chohan K (2015). Systematic review and meta-analysis of randomized controlled trials on the role of mometasone in adenoid hypertrophy in children. Int J Pediatr Otorhinolaryngol.

[9] Chorney SR, Zur KB (2021). Adenoidectomy without tonsillectomy for pediatric obstructive sleep apnea. Otolaryngol Head Neck Surg.

[10] Cuevas M (2016). Provokationstests in der Diagnostik der Rhinitis allergica.

[11] Curtis JL, Harvey DB, Willie S (2015). Causes and costs for ED visits after pediatric adenotonsillectomy. Otolaryngol Head Neck Surg.

[12] Demirbilek N, Evren C, Altun U (2015). Postadenoidectomy hemorrhage: how we do it?. Int J Clin Exp Med.

[13] Domany KA, Dana E, Tauman R (2016). Adenoidectomy for obstructive sleep apnea in children. J Clin Sleep Med.

[14] Esteller E, Villatoro JC, Aguero A (2012). Obstructive sleep apnea syndrome and growth failure. Int J Pediatr Otorhinolaryngol.

[15] Faden H, Callanan V, Pizzuto M (2016). The ubiquity ofasymptomatic respiratory viral infections in the tonsils and adenoids of children and their impact on airway obstruction. Int J Pediatr Otorhinolaryngol.

[16] Ferreira MS, Mangussi-Gomes J, Ximendes R (2018). Comparison of three different adenoidectomy techniques in children—has the conventional technique been surpassed?. Int J Pediatr Otorhinolaryngol.

[17] Galland B, Spruyt K, Dawes P (2015). Sleep disordered breathing and academic performance: a meta-analysis. Pediatrics.

[18] Garetz SL, Mitchell RB, Parker PD (2015). Quality of life and obstructive sleep apnea symptoms after pediatric adenotonsillectomy. Pediatrics.

[19] Garg A, Singh Y, Singh P (2016). Carotid artery dissection following adenoidectomy. Int J Pediatr Otorhinolaryngol.

[20] Gross IT, Bahar-Posey L (2017). Atlanto-axial subluxation after adenoidectomy. Pediatr Emer Care.

[21] Hackenberg B, Pölzl M, Matthias C (2020). Cost and value of routine histopathologic analysis after adenoidectomy and tonsillectomy. Int Arch Otorhinolaryngol.

[22] Karakas HB, Mazlumoglu MR, Simsek E (2017). The role of upper airway obstruction and snoring in the etiology of monosymptomatic nocturnal enuresis in children. Eur Arch Otorhinolaryngol.

[23] Keilmann A, Läßig AK, Pollak-Hainz A (2015). Adenoids of patients with mucopolysaccharidoses demonstrate typical alterations. Int J Pediatr Otorhinolaryngol.

[24] Khami M, Tan S, Glicksman JT (2015). Incidence and risk factors of velopharyngeal insufficiency postadenotonsillectomy. Otolaryngol Head Neck Surg.

[25] Kim JW, Kim HJ, Lee WH (2015). Comparative study for efficacy and safety of adenoidectomy according to the surgical method: a prospective multicenter study. PLoS ONE.

[26] Lautermann J, Begall K, Hilger G (2018). S2k-Leitlinie 017-004: Seromukotympanon aktueller Stand. HNO.

[27] Lee CH, Hsu WC, Chang WH (2016). Polysomnographic findings after adenotonsillectomy for obstructive sleep apnoea in obese and non-obese children: a systematic review and meta-analysis. Clin Otolaryngol.

[28] Lippert BM, Maurer J (2017). Adenotomie.

[29] Ludemann JP, Wong KK, Moxham JP (2007). Return to home, school, and sports after electrosurgical adenoidectomy: when is it safe?. J Otolaryngol.

[30] Marchica CL, Dahl JP, Raol N (2019). What’s new with tubes, tonsils, and adenoids?. Otolaryngol Clin North Am.

[31] Mikals SJ, Brigger MT (2014). Adenoidectomy as an adjuvant to primary tympanostomy tube placement: a systematic review and meta-analysis. JAMA Otolaryngol Head Neck Surg.

[32] Mitchell RB, Archer SM, Ishman SL (2019). Clinical practice guideline: tonsillectomy in children (update). Otolaryngol Head Neck Surg.

[33] Modrzynski M, Zawisza E (2007). The influence of birch pollination on the adenoid size in children with intermittent allergic rhinitis. Int J Pediatr Otorhinolaryngol.

[34] Na’ara S, Sayegh W, Nassar N (2020). Cold versus hot adenoidectomy: a prospective, randomized controlled trial. Int J Pediatr Otorhinolaryngol.

[35] Nistico L, Kreft R, Gieseke A (2011). Adenoid reservoir for pathogenic biofilm bacteria. J Clin Microbiol.

[36] Orji FT, Adiele DK, Umedum NG (2017). The clinical and radiological predictors of pulmonary hypertension in children with adenotonsillar hypertrophy. Eur Arch Otorhinolaryngol.

[37] Pagella F, Pusateri A, Canzi P (2011). The evolution of the adenoidectomy: analysis of different power-assisted techniques. Int J Immunopathol Pharmacol.

[38] Postma DS, Folsom F (2002). The case for an outpatient “approach” for all pediatric tonsillectomies and/or adenoidectomies: a 4-year review of 1419 cases at a community hospital. Otolaryngol Head Neck Surg.

[39] Randall DA (2020). Current indications for tonsillectomy and adenoidectomy. J Am Board Fam Med.

[40] Rosenfeld RM, Shin JJ, Schwartz SR (2016). Clinical practice guideline.

[41] Ryczer T, Zawadzka-Głos L, Czarnecka P (2015). Bleeding as the main complication after adenoidectomy and adenotonsillotomy. New Med.

[42] Salah M, Abdel-Aziz M, Al-Farok A (2013). Recurrent acute otitis media in infants: analysis of risk factors. Int J Pediatr Otorhinolaryngol.

[43] Schrom T (2019). Histology of adenoids. HNO.

[44] Schupper AJ, Nation J, Pransky S (2018). Adenoidectomy in children: what is the evidence and what is its role?. Curr Otorhinolaryngol Rep.

[45] Straßburg HM (2011). Schlafstörungen bei Kindern – der Stellenwert der Polysomnografie. Klin Padiatr.

[46] Stupp F, Grossi AS, Lindemann J (2020). Diagnostik und Therapie der adenotonsillären Hyperplasie im Kindesalter. HNO.

[47] Thuy ML, Rovers MM, van Staaij BK (2007). Alterations of the oropharyngeal microbial flora after adenotonsillectomy in children: a randomized controlled trial. Arch Otolaryngol Head Neck Surg.

[48] Tomkinson A, Harrison W, Owens D (2012). Postoperative hemorrhage following adenoidectomy. Laryngoscope.

[49] Türkoğlu Babakurban S, Aydın E (2016). Adenoidectomy: current approaches and review of the literature.

[50] van den Aardweg MTA, Boonacker CWB, Rovers MM (2011). Effectiveness of adenoidectomy in children with recurrent upper respiratory tract infections: open randomised controlled trial. BMJ.

[51] van den Aardweg MTA, Schilder AGM, Herkert E (2010). Adenoidectomy for otitis media in children. Cochrane Database Syst Rev.

[52] Vicini C, Eesa M, Hendawy E (2015). Powered intracapsular tonsillotomy vs. conventional extracapsular tonsillectomy for pediatric OSA: a retrospective study about efficacy, complications and quality of life. Int J Pediatr Otorhinolaryngol.

[53] Wenzel A, Königstein M, Hörmann K (2017). Standardisierte Gerinnungsanamnese vor Tonsillektomie und Adenotomie im Kindesalter. Laryngorhinootologie.

[54] Wetmore RF (2017). Surgical management of the tonsillectomy and adenoidectomy patient. World J Otorhinolaryngol Head Neck Surg.

[55] Wienke A (2021). Hno-Arzt haftet nach atemstillstand im aufwachraum: Zur verantwortlichkeit des praxisinhabers bei organisationsmängeln. Laryngorhinootologie.

[56] Wilhelm T, Hilger G, Begall K (2012). S1-Leitlinie “Adenoide Vegetationen/Rachenmandelhyperplasie”. HNO.

[57] Windfuhr JP (2019). Behandlungsfehler bei Tonsillektomie und Adenotomie und Arzthaftpflicht.

[58] Windfuhr JP (2013). Fehler und Gefahren: Tonsillektomie und andere Standard-Eingriffe. Laryngorhinootologie.

[59] Windfuhr JP, Chen YS, Remmert S (2005). Hemorrhage following tonsillectomy and adenoidectomy in 15,218 patients. Otolaryngol Head Neck Surg.

[60] Yang L, Shan Y, Wang S (2016). Endoscopic assisted adenoidectomy versus conventional curettage adenoidectomy: a meta-analysis of randomized controlled trials. SpringerPlus.

[61] Zagólski O (2010). Do diet and activity restrictions influence recovery after adenoidectomy and partial tonsillectomy?. Int J Pediatr Otorhinolaryngol.

[62] Zhu Y, Li J, Tang Y (2016). Dental arch dimensional changes after adenoidectomy or tonsillectomy in children with airway obstruction A meta-analysis and systematic review under PRISMA guidelines. Medicine.

